# Tablet-Based Apps for Phonics and Phonological Awareness: Protocol for Evidence-Based Appraisal of Content, Quality, and Usability

**DOI:** 10.2196/23921

**Published:** 2021-02-11

**Authors:** Lisa Furlong, Tanya Serry, Shane Erickson, Meg E Morris

**Affiliations:** 1 School of Education College of Arts, Social Sciences and Commerce La Trobe University Melbourne Australia; 2 School of Allied Health, Human Services and Sport College of Science, Health and Engineering La Trobe University Melbourne Australia

**Keywords:** app, appraisal, characteristics, COVID-19, health management, mHealth, mobile apps, phonics, phonological awareness, quality, reading, usability

## Abstract

**Background:**

The use of mobile apps to manage and promote health is becoming increasingly popular. Mobile apps are gaining popularity particularly in educational and interventional settings owing to their perceived advantages including support for and engagement of individuals with reading difficulties. In the context of COVID-19, the need for technology-based tools has increased. For practitioners and educators who wish to use apps in their practice or recommend apps to individuals with reading difficulties, it is challenging to identify high-quality apps in app stores.

**Objective:**

This protocol describes a systematic search, selection, and appraisal process for tablet apps targeting phonics knowledge and phonological awareness skills. This protocol aimed to (1) provide a systematic method for identifying tablet apps targeting phonics knowledge and phonological awareness skills in the Google Play Store and Apple’s App Store and (2) describe an evidence-based approach for quality appraisal of these apps by using structured tools.

**Methods:**

This protocol describes an evidence-based method guided by the PRISMA (Preferred Reporting Items for Systematic Reviews and Meta-Analyses) framework to systematically search, select, and appraise apps targeting phonics knowledge and phonological awareness skills, present in Google Play and the App Store. We intend to perform a systematic and comprehensive search and a 2-step process for screening: (1) broad screening (app titles) and (2) narrow screening (marketing descriptions). Quality appraisal of the included apps will involve two structured appraisal tools: (1) the Mobile Application Rating Scale and (2) the Appraising Apps for Reading Checklist.

**Results:**

This method will help determine the number of apps targeting phonics knowledge and phonological awareness, present on the Android and iOS platforms. The content, quality, and usability of these apps will be determined using structured appraisal tools. We have planned to conduct searches on Google Play and the App Store in January-March 2021; broad and focused screening, from April 2021; and data extraction and quality appraisal in October 2021.

**Conclusions:**

This protocol provides a basis for locating and evaluating apps targeting phonics knowledge and phonological awareness skills. This protocol will support practitioners, educators, and families to make informed decisions when purchasing apps for instructional use.

**International Registered Report Identifier (IRRID):**

PRR1-10.2196/23921

## Introduction

### Mobile Health

Mobile health (mHealth) apps are transforming health service delivery worldwide [1]. mHealth is defined as “medical and public health practice supported by mobile devices, such as mobile phones, patient monitoring devices, personal digital assistants, and other wireless devices” [1]. More than 5.2 billion individuals worldwide own a mobile device, representing approximately 67% of the world’s population [2]. The use of mobile apps to manage and promote health is becoming increasingly popular [3]. Apple recorded 45,478 health care apps in the App Store during early 2020 [4] and a total of 204 billion app downloads in 2019, which equates to US $120 billion on app-related spending [5].

In particular, educational apps have become popular [6]. Considering the enforced remote learning periods during COVID-19, the need for technology-based educational tools has increased. Educational apps are being integrated into the classroom, in speech pathology, and in educational intervention settings owing to their perceived advantages for engaged and interactive learning [7], despite limited information supporting their use [6]. The production and public use of educational apps has overtaken the research that is needed to inform their use, with hundreds of new apps being released on the app stores every day.

Worldwide, up to 40% of all children experience reading difficulties [8] potentially associated with limited early language and literacy experiences, home background [9], long absences from school [10], low socioeconomic status [11], and ineffective instruction at school [12,13]. Some children with reading difficulties have dyslexia, a specific learning disorder with impairment in word decoding, due to congenital and neurobiological differences [14]. Reading difficulties do not resolve spontaneously [15]; therefore, these individuals require timely, intensive, and explicit evidence-based interventions [11,16]. Emerging evidence suggests that mobile apps can be affordable, accessible, engaging, and effective learning tools for this population.

A systematic review by Griffith et al [17] included 11 studies evaluating outcomes related to letter knowledge, phonological awareness, letter writing, and vocabulary upon using commercially available mobile apps. Of these, 6 studies reported favorable outcomes in the app intervention group, in comparison with a control group (eg, usual classroom instruction, paper-based tasks, or the use of an app for an unrelated goal). Furthermore, Carson [18] investigated the efficacy of mobile apps for literacy among 4-year-old children with developmental language disorder and low emergent literacy skills. This between-groups pretest/posttest study revealed significant improvements in phoneme blending and segmentation and letter-sound knowledge among experimental children receiving instruction with Reading Doctor apps in comparison with control children receiving usual teacher-led emergent literacy instruction [18]. These findings have led to cautious optimism and suggest that mobile apps have the potential to improve student literacy outcomes. However, in the absence of evidence-based recommendations, it is challenging to identify high-quality mobile apps in the app stores. An initial search using terms related to two foundational literacy skills (“phonics” and “phonological awareness”) yielded approximately 2933 apps in the App Store and 4128 apps in the Google Play Store. For consumers accessing the app stores, the challenge is not only navigating through the magnitude of available apps but also being able to determine their quality, appropriateness of their content, underlying therapeutic principles, and key features of high-quality apps [17].

### The Simple View of Reading

**Figure 1:**
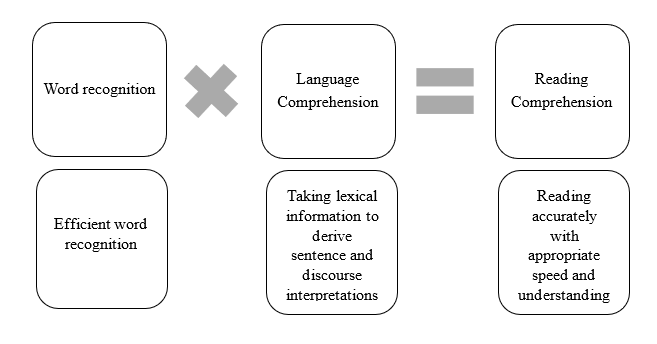
The Simple View of Reading.

The theoretical framework in this study is the empirically valid Simple View of Reading proposed by Gough and Tunmer [19-21] (Figure 1), which claims that reading comprehension is the product of two equally important components: decoding and linguistic comprehension [22]. Both components weigh equally to achieve reading comprehension [22,23].

The focus of this study is on the decoding component of the Simple View of Reading [19], which is defined as the ability to accurately segment and blend the elements within a word to enable an individual to read it. This involves 3 main skills: (1) phonics knowledge, (2) phonemic awareness skills, and (3) word-specific knowledge [24]. Phonics deals with written or printed language [24] and is defined as the ability to decode words, using knowledge of the relationships between letters (graphemes) and sounds (phonemes) [25]. Explicit teaching of systematic synthetic phonics usually occurs separately from text reading by teaching children how to build up words from graphemes. A carefully planned sequence of a small group of graphemes is taught at a time, and then blending is introduced after learning a few phoneme-grapheme correspondences [25]. Phonemic awareness skills are related to sounds in spoken language (unlike phonics, which deals with written language) [24]. Phonemic awareness skills are an essential subset of skills necessary for reading proficiency, falling under the umbrella term “phonological awareness,” which refers to “the ability to recognize and manipulate the sound properties of spoken words, such as syllables, initial sounds, rhyming parts, and phonemes” [24]. Word-specific knowledge refers to the knowledge of specific words, based on past experience [24]. This study is focused on the content, quality, and usability of apps targeting phonics knowledge and phonological awareness skills.

### Study Objectives

We provide a protocol for a systematic search, selection, and appraisal of apps targeting phonics knowledge and phonological awareness skills. This protocol addresses the following questions:

1. What tablet-based apps are currently available on the Android and iOS platforms to address phonics knowledge and phonological awareness skills?
2. What are the characteristics and features of tablet-based apps for phonics knowledge and phonological awareness skills?
3. How do the characteristics and features of tablet-based apps for phonics knowledge and phonological awareness skills compare to evidence-based principles of instruction according to the Appraising Apps for Reading Checklist (AARC)?
4. How do apps for phonics and phonological awareness rate on the Mobile Application Rating Scale (MARS) indicators of engagement, functionality, aesthetics, information quality, subjective quality, and perceived impact?

## Methods

### Study Design

Using our previously described method [26], replicated by Vaezipour et al [27], the PRISMA (Preferred Reporting Items for Systematic Reviews and Meta-Analyses) framework will be used to systematically search and select apps for quality appraisal. PRISMA is an evidence-based framework for reporting systematic reviews and meta-analyses; however, it has previously been adapted and successfully applied to review mobile health apps [27-29].

### Sources, Search Terms, and Search Strategy

Both Google Play and the App Store will be searched. These app stores have been chosen because they represent the two largest app stores, containing approximately 2.56 million and 1.85 million apps, respectively, in January-March 2020 [30]. Both app stores are linked to the most widely used operating platforms in the mobile market: Android (Google Play) and iOS (the App Store). These operating platforms accounted for approximately 99% of the global mobile market share in 2019 [31].

Google Play and the App Store will be searched using a selection of predefined search terms. These terms will be entered into the search fields of these 2 app stores, using a Samsung Galaxy Tab A (Google Play) and a 7th generation Apple iPad (the App Store). These terms (Textbox 1) have been defined in consultation with experts in the literacy domain and with consultants at Google and Apple. Preliminary searches on the app stores also contributed to these search terms, including key words obtained from potentially relevant app titles and marketing descriptions. Search results will be filtered by device to only return those items available on a tablet. The search terms include relevant synonyms and layperson terms to account for the wide variety of users accessing the app stores.

**Search terms.**

**Phonics search terms**

- Phonics
- Letters
- Letter sounds
- Alphabet
- Graphemes
- Phonemes
- Vowels
- Consonants
- Initial code
- Phonograms
- Word build
- Reading
- Spelling
- Digraphs

**Phonological awareness search terms**

- Phonological awareness
- Phonemic awareness
- Phoneme awareness
- Rhyme
- Syllables
- Segmenting sounds
- Blending sounds
- Sounding out words

### Eligibility Criteria and App Selection

The selection process aims to identify apps that can be used by individuals with reading difficulties (including children), families, educators, and interventionists to develop phonics knowledge and phonological awareness skills. For this study, the definitions previously provided for the terms “phonics” and “phonological awareness” will be used to guide decisions on app selection in addition to the following inclusion criteria: the app must run on Android or iOS, be available on a tablet, be developed for speakers of English only, be suitable for individuals of all groups (ie, no age restriction), be interactive (ie, it must not involve passive listening or watching of content), and have a word-level focus. The rationale for only including apps with a word-level focus is that explicit teaching of systematic synthetic phonics usually occurs separately from text reading by initially teaching students how to build up words from graphemes. The exclusion criteria are as follows: decodable book apps, apps for nursery rhymes, apps that teach foreign languages, apps providing only assessments, apps targeting only letter names (ie, no corresponding sounds, such as the alphabet song), sight word apps, and apps targeting only letter formation (ie, handwriting).

Our justification for focusing on only tablet-based apps is that tablet sales have exceeded those of computers worldwide owing to their increasing popularity, and students are commonly using tablets in the classroom [32]. In contrast, the use of mobile phones is not permitted in the classroom in numerous educational institutions in Australia, and an Australian policy for all government schools stipulates that mobile phones must be switched off and stored securely during the school day [33]. A review of mobile apps for childhood speech sound disorders reported that apps may be more compatible with tablets than with phones, and that few differences appear to exist between tablet and phone versions of apps, other than their layout, owing to a smaller screen size in phones [29]. These factors informed our decision to only search, select, and appraise tablet-based apps.

A 3-step process will be used to screen the apps: (1) collation of the apps for inclusion in the review, (2) broad screening, and (3) narrow screening.

#### Collation of Titles Generated by the Search

A research assistant will enter the defined search terms individually into the search field of Google Play and the App Store. Results will be filtered by tablet only. Each search term will be completed in its entirety in one sitting because app listings in the app stores constantly change depending on their relevance, popularity, and the release of new apps. A screenshot of all titles and icons of all sourced apps will be copied into a Microsoft Word document on the basis of the search term from which they were sourced. Titles will then be manually transferred to a Microsoft Excel spreadsheet on the basis of the app store in which they were located. This process is necessary as the app stores prevent copying of app data (including marketing descriptions) from their app stores. Icons will be available for reference in the Word document during screening, in case of ambiguous app titles. Duplicate app titles from different search terms from the same app store will be removed; for example, if the search terms “phonics” and “letter sounds” both yield the same app in the App Store, then one app title will be removed. When a set of app titles appears in addition to the bundle option, the bundle option will be highlighted but not be considered a separate, unique title. Non-English app titles will be removed prior to broad screening.

#### Broad Screening

Titles will be manually screened using Microsoft Excel. Two speech-language pathologists with expertise in the literacy domain will serve as reviewers during broad screening and will manually screen all titles independently and discuss the included apps for subsequent screening. Disagreements between reviewers will be resolved through discussion until consensus is achieved. If consensus cannot be achieved, a third reviewer (a speech-language pathologist with expertise in the literacy domain) will be invited to review the apps in question. A majority rule will determine the inclusion of those apps.

#### Narrow Screening

**Figure 2:**
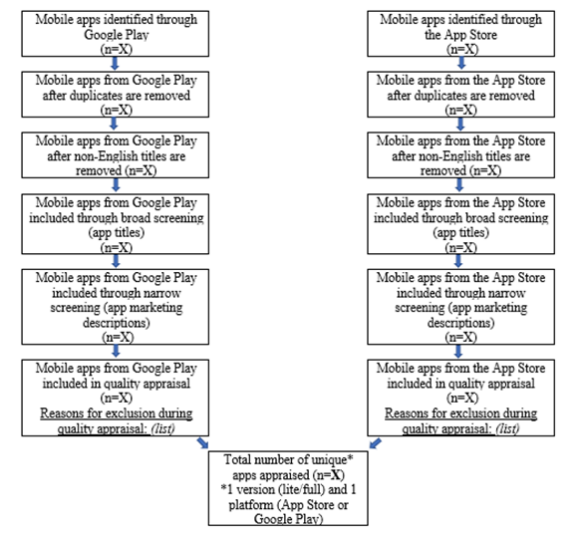
Search and selection process.

Narrow screening will involve the screening of marketing descriptions of apps included during broad screening. Marketing descriptions will be extracted from the app stores by a research assistant and entered into the same Microsoft Excel spreadsheet used in broad screening, alongside the app titles and icons. The reviewers involved in broad screening will independently review the marketing descriptions of the included apps. They will select apps on the basis of the previously described eligibility criteria and discuss those to be included in narrow screening. Similar to broad screening, reviewer disagreements will be resolved through discussion or in consultation with a third reviewer, and a majority rule will determine the inclusion of those apps. Apps finally included after narrow screening will be downloaded for quality appraisal. Figure 2 illustrates the proposed search and selection process.

### Data Extraction

For complete assessment, apps included after narrow screening will be downloaded on two devices: a 7th generation Apple iPad (10.2 inch, Wi-Fi, 128 GB) and a Samsung Galaxy Tab A (10.1 inch, Wi-Fi, 128 GB). Both tablets will operate on the most recent software version. When individual apps from one developer are available as a bundle, the bundle will be downloaded for cost efficiency. The following app classification data will be extracted from the marketing description and the app store by the first author and entered into a Microsoft Excel spreadsheet: app name and version, search terms used to identify the app, time of the latest update, app update frequency (average), number of updates, ratings for current versions, developers, number of ratings for current versions, cost (basic/upgrade), platform, bundle option, and marketing descriptions. A second reviewer will confirm the accuracy of the data extracted from 20% of the apps.

### Data Analysis

The quality of the included apps will be rated by the same reviewers involved in the screening process. Both reviewers will have clinical experience in the literacy domain and familiarity with mobile apps. Each reviewer will test the included apps for 20 minutes per app and rate each app using two structured appraisal tools: the MARS and the AARC. The MARS is a reliable tool for evaluating the quality of mobile health apps. It was developed by an expert multidisciplinary team from the Institute of Health and Biomedical Innovation, Queensland University of Technology [34]. It consists of 4 objective quality subscales using a 5-point Likert scale (engagement, functionality, aesthetics, and information quality). In addition, there are 4 questions related to subjective quality and 6 scales related to the perceived impact of the app on the user’s knowledge, intentions to change, and likelihood of actual change on the target health behavior [34].

A review of mobile apps for childhood speech sound disorders [29] reported that a key limitation of the MARS is its evaluation of an app’s potential for behavioral change; that is, how the use of the app is likely to increase or decrease the target health behavior. Based on the 4 objective indicators, an app may achieve a high total MARS score but have limited potential for behavioral change, since the subjective scales and perceived impact ratings are not included in the calculation of the total MARS score [29]. This review identified a need to evaluate various constructs related to the target health behavior objectively and comprehensively. In this study, this would involve evaluating the likelihood that use of the app would improve phonics knowledge and phonological awareness skills in accordance with the predetermined criteria. Other than tools that broadly evaluate the potential of mobile health apps to promote behavioral changes (eg, the App Behavior Change Scale [35]), there are no known or validated tools for specifically evaluating apps for phonics and phonological awareness. As this study aims to recommend apps supporting the development of phonics knowledge and phonological awareness skills, comprehensive appraisal of the content, quality, and usability of these apps is required. This appraisal should consider how the characteristics and features of these apps compare to evidence-based principles of literacy instruction and their potential to facilitate changes in phonics knowledge or phonological awareness skills.

Subsequently, the AARC—a custom-designed 19-item checklist—was developed specifically for this study. The AARC has been designed for educators and practitioners to support decision-making for the selection of apps for use in professional practice and to support the provision of evidence-based recommendations to end-users of apps intended for phonics and phonological awareness. Of the 19 AARC items, a maximum of 15 checklist items contributes to an app’s final AARC score. These 15 items are scored as 2 (yes), 1 (mostly), 0 (no), and not applicable (item excluded from the final score calculation). Based on this scoring system, the maximum total possible score is 30 (all 15 items have been rated at 2 [“yes” to all items] for an app). The AARC developers recommend interpreting the final AARC score as a percentage (ie, total points/maximum points × 100) across a continuum; that is, a high percentage would indicate a high-quality app. Furthermore, individual ratings across the AARC items can be interpreted qualitatively. For example, an app might achieve maximum scores of 2 across all 15 items except for items 4 (“Does the app allow the user to change the accent?”) and 16 (“Is the feedback or cueing therapeutically beneficial?”), for which it achieves a score of 0. In this example, the rater might consider how important it is for these features to be present in the app when the individual they are working with shares the accent of the voice present in the app and when the interventionist provides live feedback while using the app.

The AARC identifies the target of the app (ie, phonics, phonological awareness, or both) and how the skills are addressed in relation to the scope, sequence, complexity, structure, appropriateness of stimuli, delivery of instruction, practice opportunities, and feedback. The AARC also evaluates the linguistic and phonological accuracy of the app, the interactive features of the app, the potential for independent use of the app, and the likeliness of the app to improve phonics knowledge and phonological awareness skills on the basis of the rater’s subjective evaluation. This checklist was developed in consultation with academics with expertise in education (literacy), speech pathology, and mHealth.

The AARC has been piloted independently on 4 apps by two speech-language pathologists with clinical expertise in the literacy domain and mobile apps for literacy. The same overall score was obtained by both speech-language pathologists for 3 of the 4 apps. An analysis of individual AARC items revealed differences between the speech-language pathologists’ ratings. Interrater reliability was calculated on the basis of 15 items rated across 4 apps (60 items in total) for which both speech-language pathologists agreed on a rating for 54 items; therefore, the interrater reliability was 90%. The AARC is provided in Multimedia Appendix 1.

In addition to the qualitative ratings, data analysis will also include the evaluation of the total number of apps returned for each search term, the percentage of relevant apps from the yield of each search term (to guide consumers search for apps by knowing which search terms yield the most relevant results), and the correlation between consumer app ratings and ratings assigned by the speech-language pathologists involved in quality appraisal of the included apps.

## Results

Google Play and the App Store are intended to be searched in January-March 2021. Broad and narrow screening is expected to commence in April 2021. Data extraction and quality appraisal of the selected apps is expected to commence in October 2021.

## Discussion

### Principal Findings

This protocol will help identify apps that support the development of two core skills required for decoding: phonics knowledge and phonological awareness skills. Decoding is a key component of the Simple View of Reading (Figure 1) [19]—an empirically valid theoretical model of reading [20,21]. A fundamental task for beginning readers is understanding how printed language maps to their existing spoken language [23]. Automation of the process of decoding facilitates the most efficient route to reading comprehension by allowing children to focus their emerging cognitive resources to extract meaning from text [24]. These rationales support our decision to focus this study on apps for decoding, specifically those targeting phonics knowledge and phonological awareness skills.

It is difficult for consumers to identify mobile apps targeting phonics knowledge and phonological awareness skills, since >7000 apps are available on Google Play and the App Store. This protocol presents and justifies methods to systematically search, select, and appraise apps designed to target phonics knowledge and phonological awareness skills. This method includes a critical evaluation of included apps by speech-language pathologists, using structured appraisal tools. The outcomes of this method will help practitioners, families, and educators make informed decisions when selecting and recommending apps for phonics and phonological awareness. Furthermore, the outcomes of this method may support the future design and development of apps for phonics and phonological awareness by considering the characteristics and features of high-quality apps presented in this study, in collaboration with key stakeholders such as app developers, educational practitioners, literacy interventionists, and app end-users.

### Limitations

This study has some limitations of note. While the search aims to be comprehensive, the authors can only report on apps available at the time of searching and acknowledge the potential for new apps relevant to the study to be released after the search is completed. The study will only report on apps in English, thus limiting the application of this method for non-English apps. Two appraisal tools will be used to evaluate the apps: the MARS [34] and the AARC. While the AARC reportedly has good interrater reliability (90%) based on a pilot of 4 apps, it has not been evaluated extensively; therefore, the psychometric properties of validity and reliability are not available and warrant further assessment in future studies. In this study, the AARC is being used to complement the MARS by providing further information on app content and how this compares to evidence-based principles of literacy instruction, as well as the apps’ potential to bring about changes in phonics knowledge or phonological awareness skills. Another limitation of the AARC is the absence of an established quality threshold; however, considering the intended users of the AARC, app quality is expected to be determined from the total AARC score as a percentage across a continuum and by analyzing individual AARC items. Future studies on the AARC are required to establish quality descriptors based on the total AARC score. Finally, the apps will be evaluated by two speech-language pathologists rather than end-users of these apps; however, as professionals who work in the literacy domain and provide app recommendations, the outcomes may support end-user uptake of high-quality apps to support the intervention and instruction provided by educational practitioners and interventionists.

## Appendix

Multimedia Appendix 1 The Appraising Apps for Reading Checklist.

## Abbreviations

AARC: Appraising Apps for Reading Checklist
MARS: Mobile Application Rating Scale
mHealth: mobile health
PRISMA: Preferred Reporting Items for Systematic Reviews and Meta-Analyses
