# Supplementary material for: Tablet-Based Apps for Phonics and Phonological Awareness: Protocol for Evidence-Based Appraisal of Content, Quality, and Usability
Source: JMIR Res Protoc. 2021 Feb 11;10(2):e23921. doi: 10.2196/23921 (PMC7879723; doi:10.2196/23921)
Supplement: Multimedia Appendix 1 [file resprot_v10i2e23921_app1.docx]

**Appraising Apps for Reading Checklist (AARC)**

The AARC is intended for use by educators and practitioners to support decision-making around the selection of apps for use in professional practice, and to support them in providing evidence-informed recommendations to parents and guardians regarding apps for phonics and phonological awareness.

**Scoring guidelines**

The AARC consists of 19 checklist items. A maximum of 15 checklist items contribute towards an app’s final AARC score. Checklist items 1, 7, 8 and 10 do not require a quantitative rating and hence do not contribute to the AARC score. Most, or all, of the other 15 checklist items are eligible to contribute to the total AARC score. Depending on the particular app, not all questions will be relevant.

**Scoring procedure**

Questions marked with an asterisk are included in the final score. Please apply the following score to each checklist item that you rate

- Yes= 2 points
- Mostly= 1 point
- No= 0 points
- NA= removed from the final score calculation

Based on the scoring system for the AARC, the maximum total possible score is 30. This applies to an app for which all 15 items have been rated. When an item is marked ‘NA’ this is removed from the final score calculation and the maximum is interpreted in relation to the maximum possible score for the app being evaluated. The AARC developers recommend interpreting this score as a percentage, *e.g. total points/maximum points x 100* and determining overall quality based on a continuum (0-100) rather than specific cut-off points, i.e. a high percentage would be indicative of a high-quality app. Furthermore, individual ratings across the AARC items can be interpreted qualitatively.

| **Name of app being evaluated** |  |
| --- | --- |
| **Name of person evaluating the app** |  |
| **Maximum possible score for the app being evaluated** |  |
| **Final score** |  |

| **Checklist item** | **Examples or further information** | **Quantitative rating** | **Qualitative comments** |
| --- | --- | --- | --- |
| 1. What is the main target(s) of the app? | Phonics refers to the knowledge of how phonemes (speech) map onto graphemes (print). This is often referred to as knowledge of phoneme-grapheme correspondences (PGCs).  Phonological awareness is the ability to detect, isolate, and manipulate components of spoken words, including sub-lexical units such as syllables, onset and rimes, as well as individual phonemes  Consider the main target of the app. In some cases, an app may target both phonics and phonological awareness. | Phonics/phonological awareness/both |  |
| 2. Is the app linguistically accurate?***** | The way key terms are defined, or the way explanations are provided. Are the terms clearly defined? Are the terms accurately defined? | Yes/mostly/no |  |
| 3. Is the app phonologically accurate?***** | The accuracy for production of phonemes (e.g. no inserted schwas for isolated phoneme production) and the accuracy for phoneme production in relation to the chosen accent. Identify errors with PGCs, e.g. the letter ‘s’ represents /z/ (not /s/) in the word “ha**s**.” | Yes/mostly/no |  |
| 4. Does the app allow the user to change the accent?***** | Can the accent be changed? List which accents are available within the app. | Yes/no |  |
| 5. Does the app provide or recommend a systematic scope and sequence with controlled introduction of PGCs?***** | Some apps provide a predetermined scope and sequence for introducing PGCs. Other apps allow the user to determine the scope and sequence themselves. This may also include how many PGCs to target at once. For those that have a predetermined scope and sequence, consider the sequence of vowel and consonant graphemes and the variability within the sets in terms of phoneme manner, voicing and place of articulation (and the impact on phoneme blending for word reading). Identify exactly what PGCs are taught within the app (i.e. the scope); how comprehensive is this? Identify whether the app adopts a systematic approach; this means it introduces PGCs in a predetermined, logical and clearly defined sequence. | Yes/mostly/no/NA |  |
| 6. For phonological awareness apps, are a comprehensive range of skills included?***** | Apps may target any of the following phonological awareness skills: rhyme, syllables, initial phoneme identification, final phoneme identification, blending phonemes, segmenting phonemes, manipulating phonemes, etc. Consider how comprehensive the app is with the skills that are targeted. Apps targeting three or more skills should be considered *comprehensive* and rated YES. For two skills rate MOSTLY and for one skill rate NO. | Yes/mostly/no/NA |  |
| 7. Is the app’s main focus on the initial code or the extended code? | The initial code refers to common and simpler PGCs, e.g. 1:1 correspondences [such as b in the word *bat*] and 2:1 correspondences [such as *ng* in the word *hang*] for common consonant and vowel phonemes. The extended code refers to alternative spellings or less common graphemes, e.g. 3:1 and 4:1 correspondences. Examples could be the *igh* in the word *light* and the *ph* in the word *photo*. | Initial code/extended code/both/NA |  |
| 8. What type of phonics approach is used? | A synthetic phonics approach is the process in which words are broken up into the smallest units of sound (phonemes) (part to whole) for the purposes of teaching. This adopts the processes of segmenting and blending from the beginning of instruction after a few PGCs have been taught, e.g. *b-a-t* makes *bat.*  An analytic approach works from whole words to parts. It involves combining larger sound units (e.g. rimes) and encourages recognition of words as wholes before breaking them into their smaller parts, e.g. word families such as *cat, bat, mat, sat,* where the focus is on the rime *at*.  Phonics by analogy encourages the use of analogy to learn unfamiliar words, e.g. the rime of an unfamiliar word is identical to that of a known word, so the known rime is blended with the onset of the unfamiliar word, e.g. known words like *big* and *rat* are used to read the word *bat* (it has the /b/ from the known word *big* and the *at* from the known word *rat*). | Synthetic/analytic/analogy/NA |  |
| 9. Are the words used in the app appropriate for the target audience?***** | This considers the vocabulary items used in the app, e.g. are they words children would commonly encounter or use [e.g., dog, cup, jump], or are the words obscure [e.g., gut, cord, gloat]? Comment on the use of real vs. nonwords in the app, if applicable. | Yes/mostly/no/NA |  |
| 10. What level of phonotactic complexity is the app targeted at? | Consider the phonotactic complexity of the words used in the app (e.g. CVC, CCVC, CVCC, etc.); comment on whether this is comprehensive or only targeted at a specific level of phonotactic complexity. | CVC/CCVC/CVCC/  CCVCC/other |  |
| 11. Does the app provide explicit and direct instruction?***** | This means that the app provides direct and explicit instruction for the targeted skill or concept, e.g. PGCs would be taught directly and explicitly rather than children inferring PGCs from whole words. An app adopting explicit and direct instruction would not ask the user to do anything until they have been taught or shown. | Yes/mostly/no |  |
| 12. Does the app help the user work towards mastery?***** | Consider whether the app allows the user to move on without mastering previous steps or skills, i.e. can the user progress without necessarily learning the target skills? | Yes/mostly/no |  |
| 13. Does the app provide multiple opportunities for practice?***** | Consider the number of exposures or repetitions within each activity. | Yes/mostly/no |  |
| 14. Are practice tasks incorporated within a hierarchy of increasing linguistic complexity?***** | For example, the app may start with isolated PGCs and then move to single words, sentences and up to decodable readers. | Yes/mostly/no |  |
| 15. Is the app interactive?***** | Can the student interact with the app while using it? If yes, consider the level of interactivity in terms of the responses or actions required by the user when rating. Add a qualitative comment if required. | Yes/mostly/no |  |
| 16. Is the feedback or cueing therapeutically beneficial?***** | Consider the motivational impact of feedback as well as feedback for error correction, i.e. feedback for correct vs. incorrect responses. Is the feedback linked to scaffolding (e.g. the task steps down after an error is made) and is the feedback linked to progression to future tasks? | Yes/mostly/no (‘no’ should be selected for apps without feedback) |  |
| 17. Can the app be used independently by the target user?***** | Consider how much support would be needed to use the app, in relation to the target audience for the app. | Yes/mostly/no |  |
| 18. Does the app offer a free trial?***** | Is there an opportunity to “try before you buy”? | Yes/no |  |
| 19. In your opinion, is the app likely to improve phonics knowledge and/or phonological awareness skills? Why or why not?***** | Consider what gains are likely to be made as a result of using this app. | Yes/mostly/no (please comment) |  |
